# Drinking Citrus Fruit Juice Inhibits Vascular Remodeling in Cuff-Induced Vascular Injury Mouse Model

**DOI:** 10.1371/journal.pone.0117616

**Published:** 2015-02-18

**Authors:** Arika Ohnishi, Rie Asayama, Masaki Mogi, Hirotomo Nakaoka, Harumi Kan-no, Kana Tsukuda, Toshiyuki Chisaka, Xiao-Li Wang, Hui-Yu Bai, Bao-Shuai Shan, Masayoshi Kukida, Jun Iwanami, Masatsugu Horiuchi

**Affiliations:** 1 Department of Molecular Cardiovascular Biology and Pharmacology, Ehime University, Graduate School of Medicine, Tohon, Ehime, Japan; 2 Department of Pediatrics, Ehime University, Graduate School of Medicine, Tohon, Ehime, Japan; 3 Department of Cardiology, Pulmonology, Hypertension and Nephrology, Ehime University, Graduate School of Medicine, Tohon, Ehime, Japan

## Abstract

Citrus fruits are thought to have inhibitory effects on oxidative stress, thereby attenuating the onset and progression of cancer and cardiovascular disease; however, there are few reports assessing their effect on vascular remodeling. Here, we investigated the effect of drinking the juice of two different citrus fruits on vascular neointima formation using a cuff-induced vascular injury mouse model. Male C57BL6 mice were divided into five groups as follows: 1) Control (water) (C), 2) 10% *Citrus unshiu* (CU) juice (CU10), 3) 40% CU juice (CU40), 4) 10% *Citrus iyo* (CI) juice (CI10), and 5) 40% CI juice (CI40). After drinking them for 2 weeks from 8 weeks of age, cuff injury was induced by polyethylene cuff placement around the femoral artery. Neointima formation was significantly attenuated in CU40, CI10 and CI40 compared with C; however, no remarkable preventive effect was observed in CU10. The increases in levels of various inflammatory markers including cytokines such as monocyte chemotactic protein-1, interleukin-6 (IL-6), IL-1β, and tumor necrosis factor-α in response to vascular injury did not differ significantly between C, CU10 and CI10. The increases in cell proliferation and superoxide anion production were markedly attenuated in CI10, but not in CU10 compared with C. The increase in phosphorylated ERK expression was markedly attenuated both in CU10 and CI10 without significant difference between CU10 and CI10. Accumulation of immune cells did not differ between CU10 and CI10. These results indicate that drinking citrus fruit juice attenuates vascular remodeling partly via a reduction of oxidative stress. Interestingly, the preventive efficacy on neointima formation was stronger in CI than in CU at least in part due to more prominent inhibitory effects on oxidative stress by CI.

## Introduction

Dietary habits are closely related to most lifestyle-related diseases such as cardiovascular disease (CVD). Therefore, dietary modification is one of the cornerstones for prevention of the onset of CVD. High consumption of fruit is reported to be beneficial for reduction of cardiovascular mortality [1], prevention of ischemic heart disease [2] and stroke [3], and blood pressure lowering [4,5,6,7]. Among them, eating citrus fruits and/or drinking their juice have preventive effects on the pathophysiology of ischemic stroke [8] and CVD etc. [9]

Citrus fruits are one of the most popular and highly consumed type of fruit in the United States and Japan. *Citrus unshiu* (also known as Mandarin orange or Mikan) is the most popular citrus fruit and ingredient of fruit juice in Japan. Moreover, *Citrus iyo* (also known as iyokan) is the second most widely produced citrus fruit in Japan. Recently, the effects of consumption of *Citrus unshiu* on cognitive function [10], nitric oxide production [11] and allergic rhinitis [12] have been reported, suggesting that *Citrus unshiu* intake could have beneficial effects on the pathophysiology of cardiovascular disease.

Buscemi et al. recently demonstrated that drinking red orange juice for seven days ameliorated endothelial dysfunction and reduced inflammation in non-diabetic subjects with increased cardiovascular risk [13]. Moreover, Codoñer-Franch et al. reported that drinking mandarin juice showed an anti-oxidant effect in children [14,15] and rats [16]. However, red orange juice is not popular in Japan, and the effect of drinking citrus juice on vascular function has never been investigated using animal models. Therefore, we investigated the effect of drinking citrus juice on vascular remodeling using a cuff-induced vascular injury model in mice, and we also examined the possible difference in vascular protective effects between *Citrus unshiu* and *Citrus iyo*.

## Methods

All procedures were performed in accordance with the National Institutes of Health Guide for the Care and Use of Laboratory Animals, and reviewed and approved by the Animal Studies Committee of Ehime University.

### Citrus Fruit Juice Preparation

The juice of *Citrus unshiu* (CU) and *Citrus iyo* (CI) was prepared and provided by Ehime Beverage Inc. (Ehime, Japan). Citrus juice was prepared as follows. Juice was extracted from washed fresh CU or CI by in-line juice extractor (John Bean Technologies Corp., Chicago, IL). Using a hydro-cyclone between the procedures of extractors and finishers, the defects such as embryonic seeds were removed. After treatment with finishers, juice was centrifuged at 7,500 x g to remove the part of insoluble solids. The juice was filled into bottles followed pasteurization at 96°C for 30 seconds and cooling. The bottles were kept in the cold storage at -18°C before use.

### Animals and Treatment

Male C57BL6 mice (CLEA, Tokyo, Japan) were divided into five groups as follows: 1) Control (water) (C), 2) 10% CU (CU10), 3) 40% CU (CU40), 4) 10% CI (CI10), and 5) 40% CI (CI40). The animals were housed in a room in which lighting was controlled (12 hours on and 12 hours off), and room temperature was kept at 25°C. They were given a standard diet (MF, Oriental Yeast Co., Ltd., Tokyo, Japan). After drinking juice ad libitum for 2 weeks from 8 weeks of age, cuff injury was induced by polyethylene cuff placement around the femoral artery. After 2 weeks of drinking citrus fruit juice, systolic blood pressure was measured by the tail-cuff method (MK-2000ST, Muromachi Kikai, Co. Ltd., Tokyo, Japan) as described previously [17].

### Cuff-induced Vascular Injury Model

Adult male C57BL/6J mice (10 weeks of age) were used in this study. Inflammatory cuff injury was induced by polyethylene cuff placement around the femoral artery under anesthesia with intraperitoneal injection of 60 mg/kg pentobarbital sodium in saline, and morphometric analysis to measure the neointimal area was performed as described previously [18,19].

### Immunohistochemical Staining

Formalin-fixed, paraffin-embedded sections were prepared using femoral artery 7 days after cuff placement. Endogenous peroxidase was blocked by incubation in 3% H_2_O_2_ for 15 min, and nonspecific protein binding was blocked by incubation for 10 min in Blocking Reagent (Nichirei Bioscience Inc., Tokyo, Japan). The sections were incubated overnight at 4°C with the primary antibody, anti-proliferating cell nuclear antigen (PCNA) antibody (Novocastra Laboratories, Ltd., Newcastle upon Tyne, UK), and phosphorylated extracellular signal-regulated kinase (ERK) and total ERK antibodies (Cell Signaling Technology, Pickering, ON) and Alexa Fluor 488-conjugated F4/80 (BioLegend Inc., San Diego, CA) and R-PE-conjugated LY-6G/-6C (Hycult Biotech Inc., Plymouth Meeting, PA). Antibody binding was visualized by followings: 1) a Zeiss Axioskop2 microscope (Carl Zeiss, Oberkochen, Germany) equipped with a computer-based imaging system for 3, 3’-diaminobenzidine (DAB) staining using a detection kit, Histofine (Nichirei Bioscience Inc.) for PCNA and ERK staining, and 2) a fluorescence microscope (Keyence BZ-9000, Osaka, Japan) equipped with a computer-based imaging system for immunofluorescent staining.

### Real Time RT-PCR

Total RNA was extracted from a pool of four different femoral arteries. Real-time quantitative reverse-transcription polymerase chain reaction (PCR) was performed with a SYBR Premix Ex Taq (Takara Bio Inc., Shiga, Japan). PCR primers were as follows: MCP-1, 5’-TTAACGCCCCACTCACCTGCTG-3’ (forward) and 5’-GCTTCTTTGGGACACCTGCTGC-3’ (reverse); tumor necrosis factor-α (TNF-α), 5’-CGAGTGACAAGCCTGTAGCC-3’ (forward) and 5’-GGTGAGGAGCACGATGTCG-3’ (reverse); IL-6, 5’-CCACTTCACAAGTCGGAGGCTTA-3’ (forward) and 5’-GCAAGTGCATCATCGTTGTTCATAC-3’ (reverse); interleukin-1β (IL-1β), 5’-TCCAGGATGAGGACATGAGCAC-3’ (forward) and 5’-GAACGTCACACACCAGCAGGTTA-3’ (reverse); glyceraldehyde-3-phosphate dehydrogenase (GAPDH), 5’-ATGTAGGCCATGAGGTCCAC-3’ (forward) and 5’-TGCGACTTCAACAGCAACTC-3’ (reverse).

### Dihydroethidium Staining

Superoxide generation in cryostat frozen sections was evaluated using fluorogenic dihydroethidium (5 μmol/L), as described previously [20]. Intensity of fluorescence was analyzed and quantified using computer imaging software (Densitograph, ATTO Corp., Tokyo, Japan).

### Immunoblot Analysis

Femoral arteries were obtained 7 days after cuff placement and the adventitia was carefully removed. The extracted proteins from the femoral arteries were subjected to SDS-PAGE and immunoblotted with rabbit anti-phosphorylated ERK and total ERK antibodies (Cell Signaling Technology). The bands of proteins were visualized using an enhanced chemiluminescence system (GE Healthcare, Buckinghamshire, England).

### Statistical Analysis

All values are expressed as mean ± S.D. in the text and figures. Data were evaluated by ANOVA. If a statistically significant effect was found, post hoc analysis was performed to detect the difference between the groups. Values of *P* < 0.05 were considered to be statistically significant.

## Results

### Inhibitory Effect of Citrus Fruits on Neointima Formation

**Fig 1:**
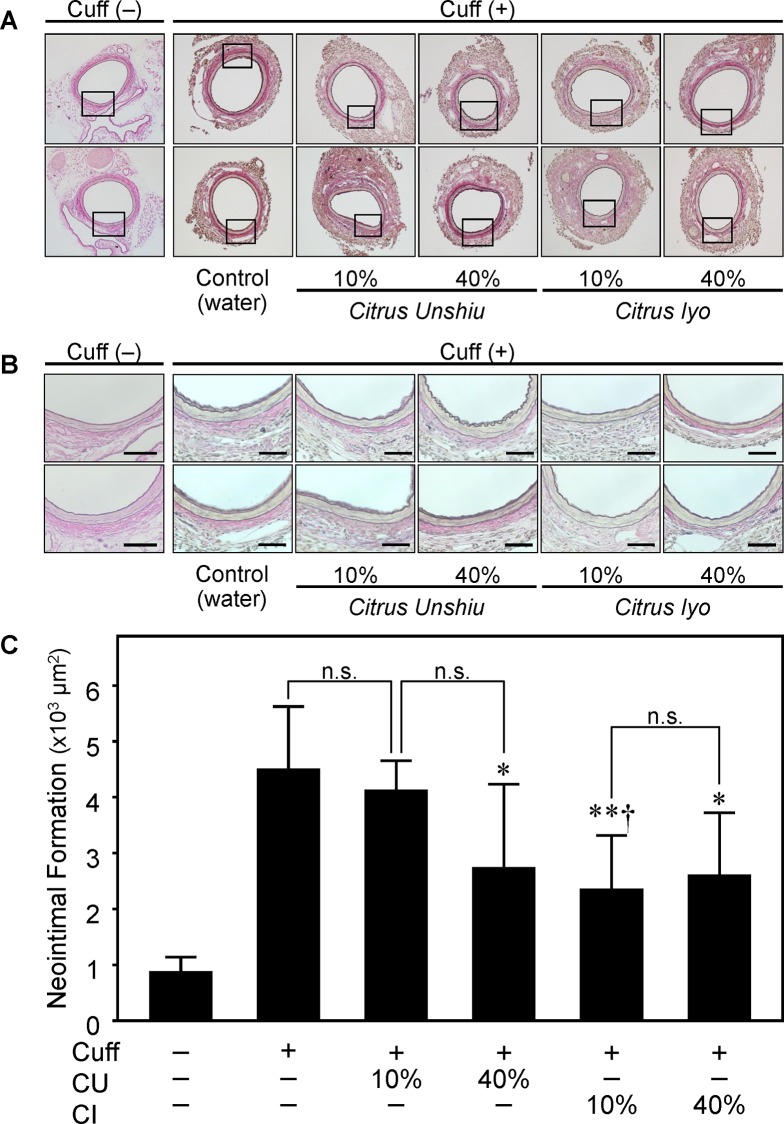
Effect of drinking citrus fruit juice on neointima formation. Male C57BL6 mice were divided into five groups as follows: 1) Control (water) (C), 2) 10% *Citrus unshiu* (CU) juice (CU10), 3) 40% CU juice (CU40), 4) 10% *Citrus iyo* (CI) juice (CI10), and 5) 40% CI juice (CI40). After drinking them for 2 weeks from 8 weeks of age, cuff injury was induced by polyethylene cuff placement around the femoral artery. Samples were prepared from cuffed-femoral arteries of C57BL/6J mice as described in Methods. A, Representative photos of neointimal area in cross-sections of femoral artery with elastic van Gieson staining 14 days after cuff placement at 100x magnification. B, Higher magnified photos at 400x magnification described as squares in Figure A. Scale bars show 50 μm in each photo. C, Quantitative analysis of neointimal area in injured femoral artery. Values are mean ± SEM (n = 6 for Cuff (-), n = 8 for other groups). *p<0.05, **p<0.01 vs. Cuff (+) Control, †p<0.05 vs. administration of juice of different citrus fruit at same %.

We examined the effects of drinking citrus fruit juice on neointima formation 14 days after polyethylene cuff placement around the femoral artery, and observed that drinking citrus fruit juice attenuated neointima formation (**Fig. 1A** and **1B**) without a change in body weight, blood glucose or blood pressure (**S1 Fig**). Neointima formation was significantly attenuated in CU40, CI10 and CI40 compared with C; however, no remarkable preventive effect was observed in CU10. During the experimental period, no significant change in fluid intake was observed in each group (**S2 Fig**). To assess the more detailed mechanism of the differences between CU and CI in terms of the inhibitory effect on neointimal formation and cell proliferation, we focused on three groups: control, CU10 and CI10.

### Inhibitory Effect of Citrus Fruits on Cell Proliferation

**Fig 2:**
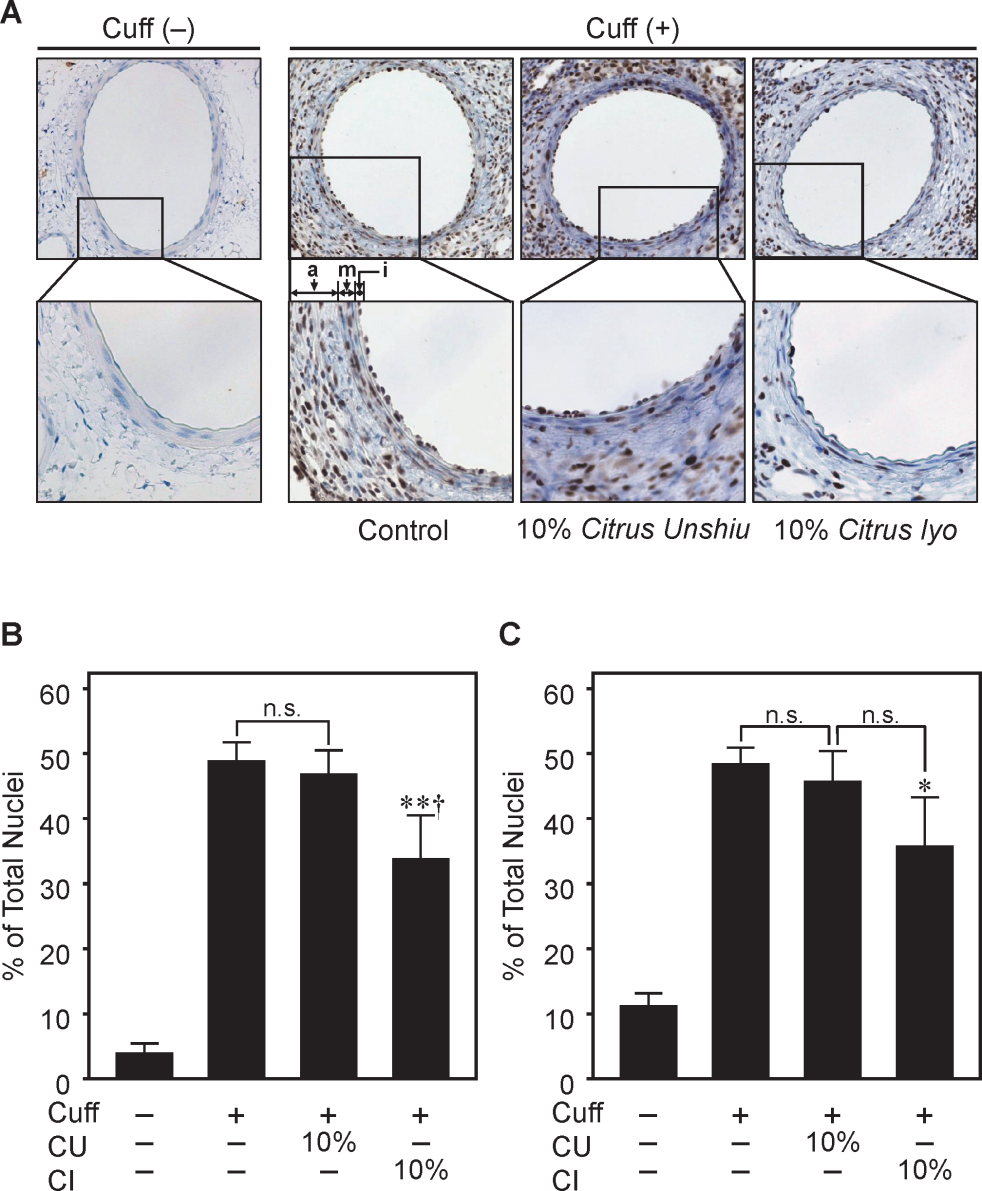
Effect of drinking citrus fruit juice on cell proliferation in injured femoral artery after cuff placement. Representative photos of injured femoral artery in cross-sections after PCNA staining (A) (200x and 400x magnification) and PCNA labeling index in intima (B) and media (C). Higher magnified photos described as squares in Figure A. Cuff placement around the femoral artery was performed, and PCNA was detected as described in Methods. Cell proliferation was measured as the ratio of PCNA-positive nuclei to total nuclei in the femoral artery 7 days after cuff placement. CU; *Citrus unshiu* juice, CI; *Citrus iyo* juice. a; adventitia, m; media, i; intima. Values are mean ± SEM (n = 6 for Cuff (-), n = 4 for other groups). *p<0.05, **p<0.01 vs. Cuff (+) Control, †p<0.05 vs. administration of juice of different citrus fruit at same %.

We observed that drinking citrus fruit juice decreased the neointima area, with a decrease in PCNA labeling index in both the intima and media (**Fig. 2A** and **2B**). The increase in cell proliferation after cuff-placement was markedly attenuated in CI10, but not in CU10 compared with C.

### Inflammatory Cytokine Levels Were Not Attenuated by Citrus Fruits

**Fig 3:**
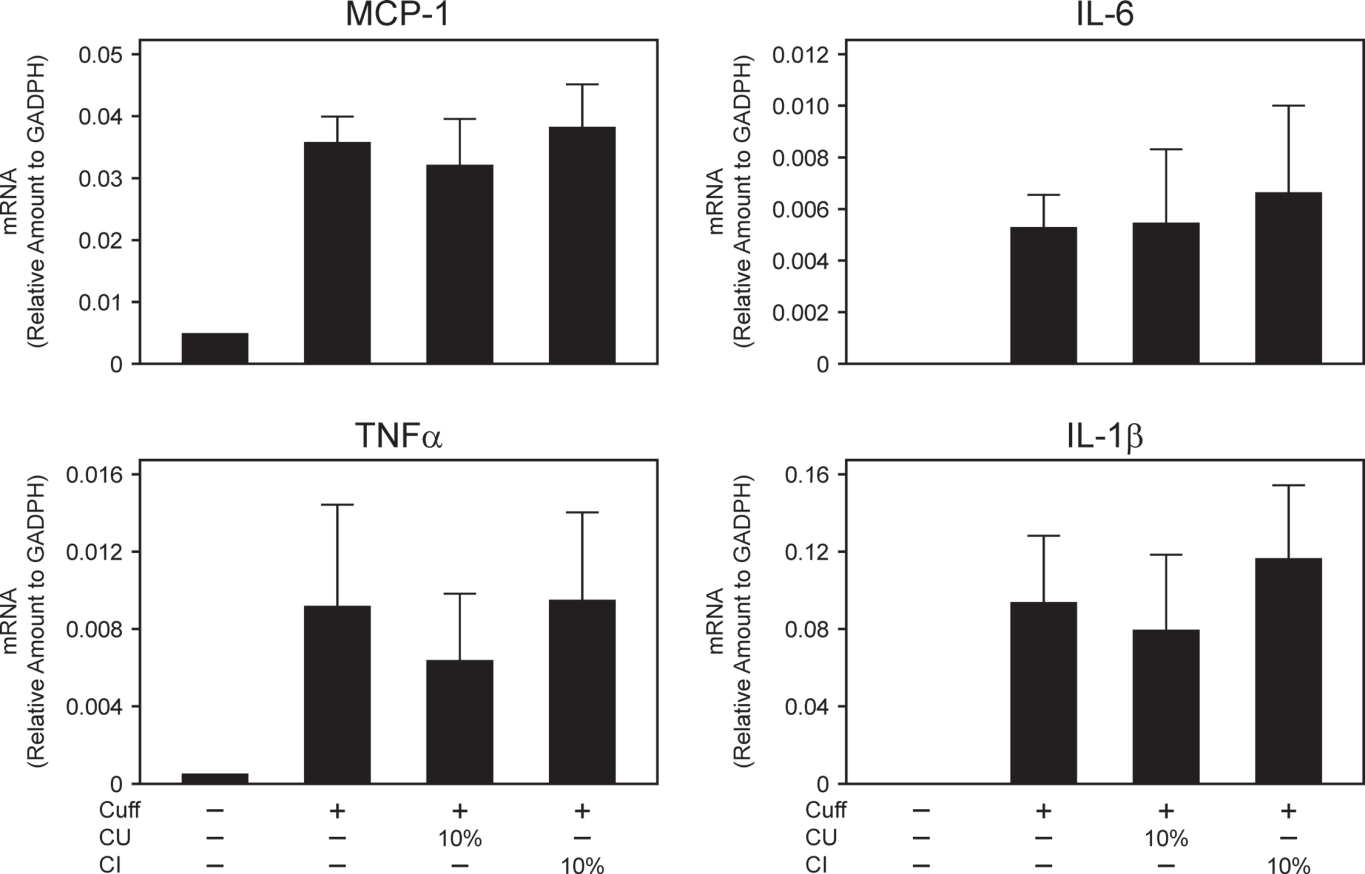
Effect of drinking citrus fruit juice on inflammatory cytokines. Expression of TNF-α, MCP-1, IL-6, and IL-1β determined by real-time quantitative RT-PCR in femoral artery 7 days after cuff placement. Tissue samples were prepared from cuffed arteries 7 days after operation. CU; *Citrus unshiu* juice, CI; *Citrus iyo* juice. Values are mean ± SEM (n = 4 for each group).

We assessed the effect of drinking citrus fruit juice on mRNA levels of inflammatory cytokines in the femoral artery 7 days after cuff placement (**Fig. 3**). The increases in various inflammatory cytokines such as monocyte chemotactic protein-1, interleukin-6 (IL-6), IL-1β and tumor necrosis factor-α in response to vascular injury did not differ significantly among C, CU10 and CI10.

### Inhibitory Effect of Citrus Fruits on Superoxide Anion Production

**Fig 4:**
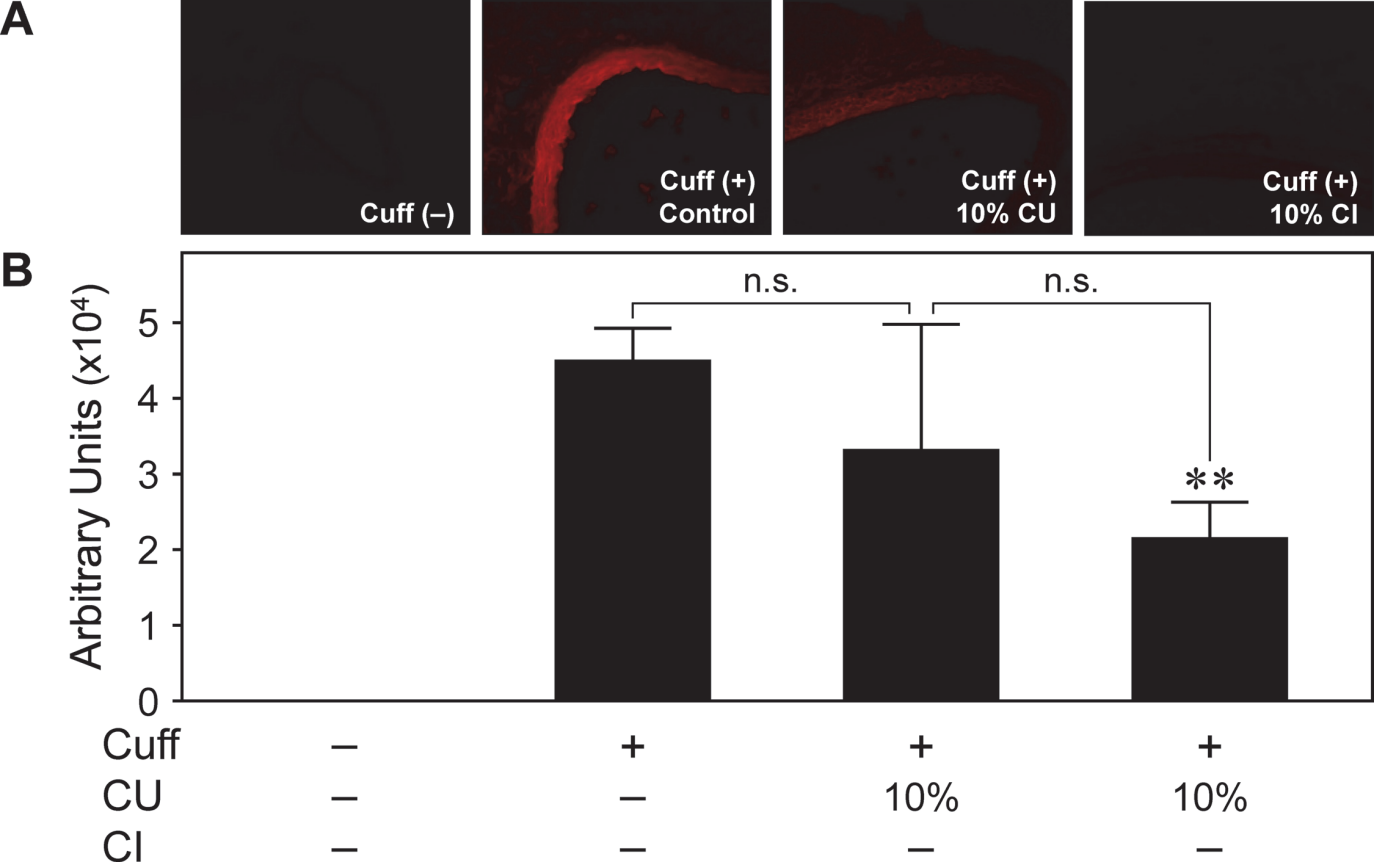
Effect of drinking citrus fruit juice on superoxide anion production induced in injured artery. Tissue samples were prepared from cuffed arteries 7 days after operation. Representative photos of injured femoral artery in cross-sections after dihydroethidium staining (A) and fluorescence intensity in intima and media (B). CU; *Citrus unshiu* juice, CI; *Citrus iyo* juice. **p<0.01 vs. Cuff (+) Control. Values are mean ± SEM (n = 7 to 8 for each group).

Next, we examined the effect of drinking citrus fruit juice on superoxide anion production in the femoral artery 7 days after cuff placement (**Fig. 4A** and **4B**). The increase in superoxide anion production determined by dihydroethidium staining was markedly attenuated in CI10, but not in CU10 compared with C.

### Inhibitory Effect of Citrus Fruits on ERK Activation

**Fig 5:**
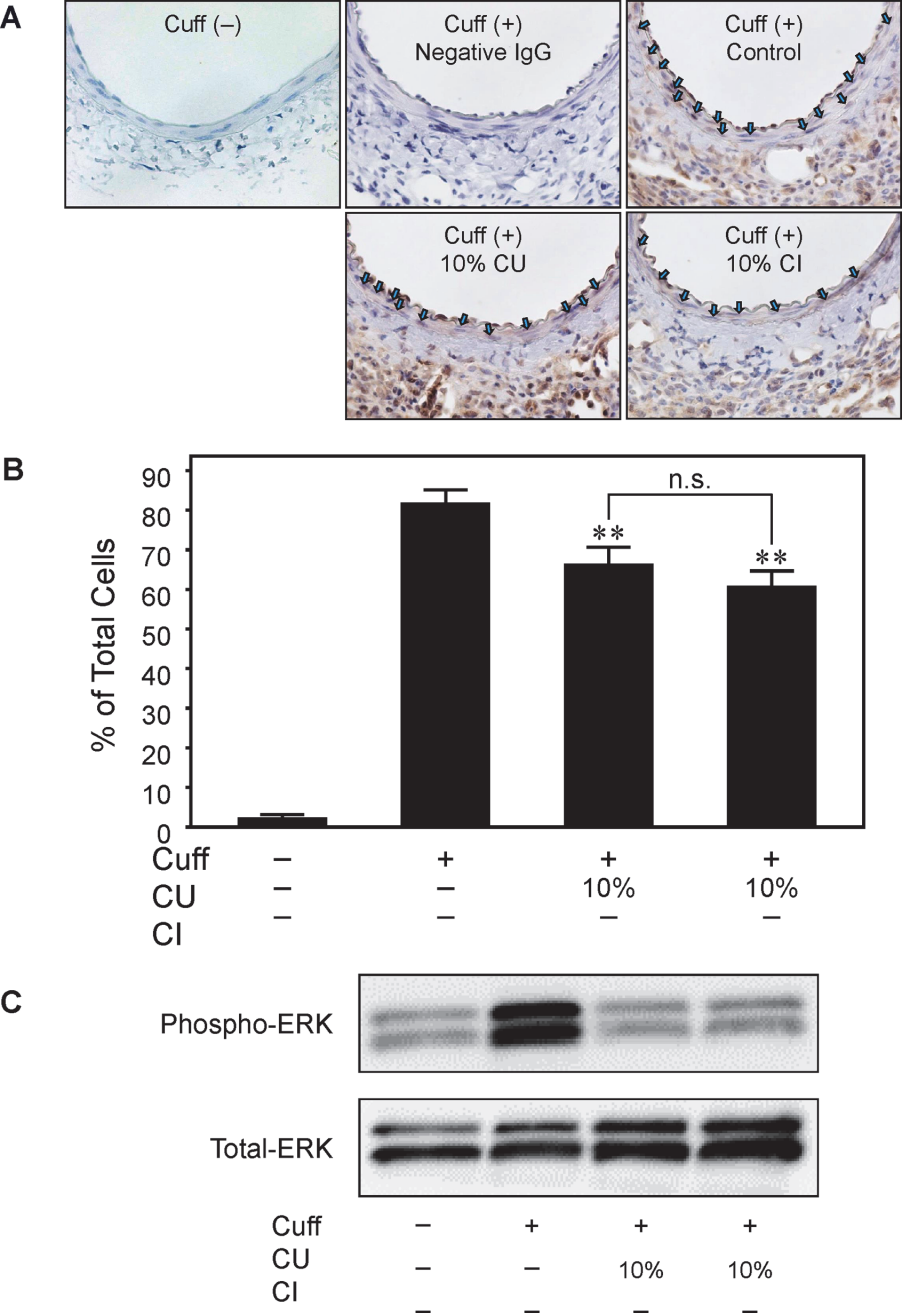
Effect of drinking citrus fruit juice on ERK activation. Comparison of ERK activation in injured femoral artery 7 days after cuff placement. A, Representative photos of injured femoral artery in cross-sections after phospho-ERK staining. Blue arrows indicate phosphor-ERK positive nuclei. B, Quantitative analysis of positive stained cell ratio in neointima. Values are mean ± SEM (n = 5 for Cuff (-), n = 5 for Cuff (+) Control and CU10% group and n = 3 for CI10% group). **p<0.01 vs. Cuff (+) Control. C, Representative immunoblots of ERK in pooled samples are shown. CU; *Citrus unshiu* juice, CI; *Citrus iyo* juice.

We investigated ERK activation in the injured artery 7 days after cuff placement (**Fig. 5**). The increase in expression of phosphorylated ERK determined by immunohistochemical staining and immunoblot using anti-phosphorylated ERK antibody. The increase in phosphorylated ERK positive cells in neointima determined by immunohistochemical staining was markedly attenuated both in CU10 and CI10 compared with C (**Fig. 5A and 5B**). Consistent with these immunohistochemical　results, ERK activity determined with Western blotting was markedly attenuated in CI10 and CU10 compared with C (**Fig. 5C**). However, significant difference in the inhibitory effects between CU10 and CI10 on ERK activation was not observed.

### Effect of Citrus Fruits on Immune Cells

**Fig 6:**
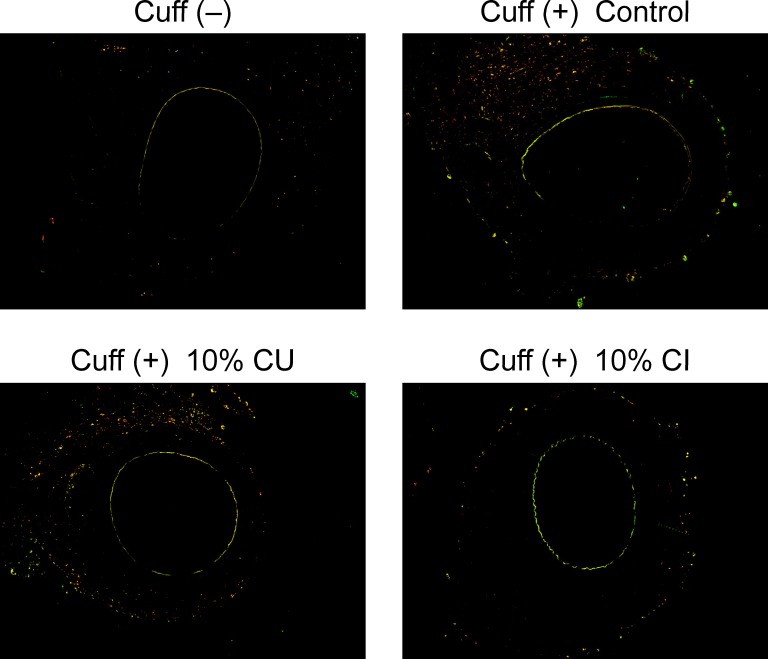
Effect of drinking citrus fruit juice on immune cell infiltration. Comparison of filtration of macrophage and neutrophils in injured femoral artery 7 days after cuff placement. Representative photos of injured femoral artery in cross-sections after immunofluorescent staining using antibodies against a macrophage marker, F4/80 and a neutrophil marker, LY-6G/-6C. CU; *Citrus unshiu* juice, CI; *Citrus iyo* juice.

We investigated the effect immune cells in the injured artery 7 days after cuff placement using antibodies against a macrophage marker, F4/80 and a neutrophil marker, LY-6G/-6C (**Fig. 6**). Macrophages and neutrophils accumulated around adventitia of cuff-injured arteries. Increase in these accumulations was attenuated in CI10 and CU10 compared with C. However, no remarkable change was observed between CU10 and CI10.

## Discussion

These results indicate that drinking citrus fruit juice attenuated vascular remodeling partly via a reduction of oxidative stress and proliferative signaling. Interestingly, the inhibitory effect on neointima formation was stronger in CI compared with CU. Previous reports about the effects of consumption of citrus drink have focused mainly on cancer prevention due to its anti-oxidant effect [21,22,23]. Cancer preventive effects of CU have also been reported [24,25,26]. However, to our knowledge, the preventive effect of CU and CI on vascular remodeling has not been previously reported.

Intake of orange juice has been reported to reduce oxidative stress and inflammation. Drinking orange juice with a high-fat and high-carbohydrate meal prevented the marked increases in reactive oxygen species (ROS) generation and other inflammatory indexes, with attenuation of the increase in Toll-like receptors (TLR) 2, TLR4 and suppressor of cytokine signaling (SOCS)-3 expression in normal-weight subjects [27]. Moreover, drinking orange juice reduced serum IL-1β and IL-6 levels in healthy human volunteers [28]. In our experiment, there was no remarkable difference in pro-inflammatory cytokine levels between mice with and without drinking of citrus juice. In human studies, samples are prepared from blood or urine; therefore, there are study limitations in assessing the effect of drinking citrus juice on local tissue levels in humans. Animal models could provide more mechanistic insights into the possible role of the anti-oxidative stress or anti-proliferative effects of drinking citrus fruit juice in the prevention of vascular remodeling at the local tissue level.

We observed a difference in the preventive effects on vascular remodeling between CU and CI. CI exhibited a greater preventive effect on superoxide anion production and proliferative response compared with CU. **Table 1** shows the comparison of contents between CU and CI juice by calculated values based on the Standard Tables of Food Composition in Japan [29]. Retinol equivalent (RE), β-carotene and β-cryptoxanthin levels are significantly higher in CU juice. In contrast, flavanone concentrations especially in eriocitrin, narirutin and hesperidin are remarkably higher in CI juice as shown in **Table 2** [30]. Morand et al. demonstrated that the intake of hesperidin administered as orange juice or as pure compound induced positive effects on vascular protection [31]. Recently, Xu et al. reported that naringenin, an aglycone and a metabolite of naringin, exhibited antioxidant activity on angiotensin II-treated vascular smooth muscle cells and balloon injured rat carotid arteries [32]. Although naringin is not detected in both citrus juices, an increase level in flavone could prevent vascular remodeling. Moreover, *Citrus iyo* contains relatively high levels of limonoids such as limonin, nomilin and nomilinic acid, which contribute to bitterness [33] and are expected to prevent cancer, activate detoxification enzymes, attenuate hypercholesterolemia and decrease acylglycerol [34]. The possible difference in the inhibitory effect on neointima formation between CI and CU could be explained by the higher limonoid levels in CI than in CU. We are now investigating the detailed mechanism of their difference, focusing on their compositions in in vitro studies using vascular smooth muscle cells, since it is not easy to study the effect of limonoids on vascular remodeling in vivo.

**Table 1:** Comparison of contents and ingredients between *Citrus unshiu* and *Citrus iyo* juice.

/100 g	Citrus unshiu	Citrus iyo
Calories (kcal)	41	44
Water content (g)	88.5	88.6
Protein (g)	0.5	0.6
Total fat (g)	0.1	0.1
Total carbohydrate (g)	10.6	10.5
Mineral content (g)	0.3	0.4
Sodium (mg)	1	2
Potassium (mg)	130	163
Calcium (mg)	8	15
Magnesium (mg)	8	12
Phosphorus (mg)	11	15
Iron (mg)	0.2	0.2
Zinc (mg)	Trivial	0.1
Copper (mg)	0.02	0.03
Manganese (mg)	0.03	0.06
Iodine (μg)	1	-
Selenium (μg)	Trivial	-
Chromium (μg)	1	-
Molybdenum (μg)	Trivial	-
Retinol (μg)	0	0
α-carotene (μg)	2	0
β-carotene (μg)	53	13
β- cryptoxanthin (μg)	740	106
Retinol equivalent (μg)	35	6
β-carotene equivalent (μg)	420	66
Vitamin D (μg)	0	0
α-tocopherol (mg)	0.2	0.1
β-tocopherol (mg)	0	0
γ-tocopherol (mg)	0	0
δ-tocopherol (mg)	0	0
Vitamin K (μg)	0	0
Vitamin B1 (mg)	0.06	0.05
Vitamin B2 (mg)	0.01	0.03
Niacin (mg)	0.2	0.3
Vitamin B6 (mg)	0.03	0.06
Vitamin B12 (μg)	0	0
Folate (μg)	15	16
Pantothenic acid (mg)	0.14	0.31
Biotin (μg)	0.3	-
Vitamin C (mg)	29	31
Saturated fat (g)	0.01	-
Monosaturated fat (g)	0.02	-
Polysaturated fat (g)	0.01	-
Cholesterol (mg)	0	0
Soluble dietary fiber (g)	0	0
Insoluble dietary fiber (g)	0	0
Total dietary fiber (g)	0	0
Salt equivalent (g)	0	0

**Table 2:** Comparison of flavonoid concentrations between *Citrus unshiu* and *Citrus iyo* juice.

(mg)/100 g	Citrus unshiu	Citrus iyo
Flavanone		
Eriocitrin (ERC)	0.2	2.1
Neoeriocitrin (NER)	0	0
Narirutin (NRT)	15.4	125
Naringin (NRG)	0	0
Hesperidin (HSP)	8.7	178
Neohesperidin (NHP)	0	0
Neoponcirin (NPO)	0	3
Poncirin (PON)	0	0
Flavone		
Rutin (RTN)	0	0
Isorhoifolin (IRF)	0	0
Rhoifolin (RFN)	0	0
Diosmin (DSM)	1.4	0
Neodiosmin (NDM)	0	0
Polymethoxylated flavone		
Sinensetin (SNT)	0	0
Nobiletin (NOB)	0	0
Tangeretin (TNG)	0	0
Heptamethoxyflavone (HPM)	0	0

We evaluated the immune cell infiltration in this model as shown in **Fig. 6**. Treatment with CU and CI prevented these accumulations. We have also investigated the effect of macrophage depletion using clodronate to evaluate immune cell function. Treatment with clodronate (0.1 μL) just before cuff placement did not show a remarkable effect on neointimal formation (data not shown); however, administration of clodronate resulted in high mortality due to the failure of wound healing via reduction of macrophages. Therefore, it is hard to conclude the effect of CU or CI on vascular remodeling via affecting immune cell function. Further investigation is necessary to prove the detailed mechanism.

In spite of the beneficial effects of drinking citrus juice in terms of protection against vascular injury, the possible risk of increased incidence of type 2 diabetes mellitus (T2DM) has to be taken in account. Although total fruit consumption is not consistently associated with risk of T2DM, higher fruit juice consumption was associated with a higher risk [35]. Sugar-sweetened beverages, but not 100% citrus juice, are associated with the development of metabolic syndrome and T2DM [36]. Therefore, the appropriate amount of citrus drink without sugar per day to prevent metabolic disorders such as T2DM and obesity in humans should be assessed.

In summary, our findings support the notion that drinking citrus fruit juices has a beneficial effect on vascular remodeling, at least due to their inhibitory effects on superoxide anion production, cell proliferation, and phosphorylated ERK expression. We can expect that drinking citrus fruit juice, especially CI, would have beneficial effects of preventing vascular diseases such as atherosclerosis.

## Supporting Information

S1 Fig Effect of drinking citrus fruits on body weight, blood glucose and blood pressure. C; Control, CU; Citrus Unshiu, CI; Citrus Iyo. Values are mean ± SEM of 6 mice.
(PDF)

S2 Fig Effect of drinking citrus fruits on fluid intake. CU; Citrus Unshiu, CI; Citrus Iyo.
(PDF)
