# Supplementary material for: Drinking Citrus Fruit Juice Inhibits Vascular Remodeling in Cuff-Induced Vascular Injury Mouse Model
Source: PLoS One. 2015 Feb 18;10(2):e0117616. doi: 10.1371/journal.pone.0117616 (PMC4334235; doi:10.1371/journal.pone.0117616)
Supplement: S1 Fig — C; Control, CU; Citrus Unshiu, CI; Citrus Iyo. Values are mean ± SEM of 6 mice. (PDF) [file pone.0117616.s001.pdf]

	C		10% CU		40% CU		10% CI		40% CI	
Treatment	Before	After	Before	After	Before	After	Before	After	Before	After
Body Weight (g)	22.4 ±0.7	24.0 ±0.7	22.6 ±0.8	24.2 ±1.2	23.2 ±0.6	24.7 ±0.8	22.7 ±0.5	24.5 ±0.8	23.4 ±0.9	24.4 ±0.9
Blood Glucose (mg/dL)	185.3 ±23.9	174.8 ±30.0	161.0 ±13.6	155.8 ±21.5	173.5 ±24.3	175.2 ±18.0	138.3 ±26.6	171.2 ±27.5	173.2 ±38.2	157.7 ±6.1
Blood Pressure (mmHg)	104.7 ±6.4	101.8 ±11.8	92.8 ±6.9	118.2 ±14.4	96.8 ±6.9	100.8 ±7.7	92.3 ±2.6	104.2 ±5.3	100.5 ±12.0	101.8 ±9.8
