# Supplementary figures and images for: Drinking Citrus Fruit Juice Inhibits Vascular Remodeling in Cuff-Induced Vascular Injury Mouse Model
Source: PLoS One. 2015 Feb 18;10(2):e0117616. doi: 10.1371/journal.pone.0117616 (PMC4334235; doi:10.1371/journal.pone.0117616)

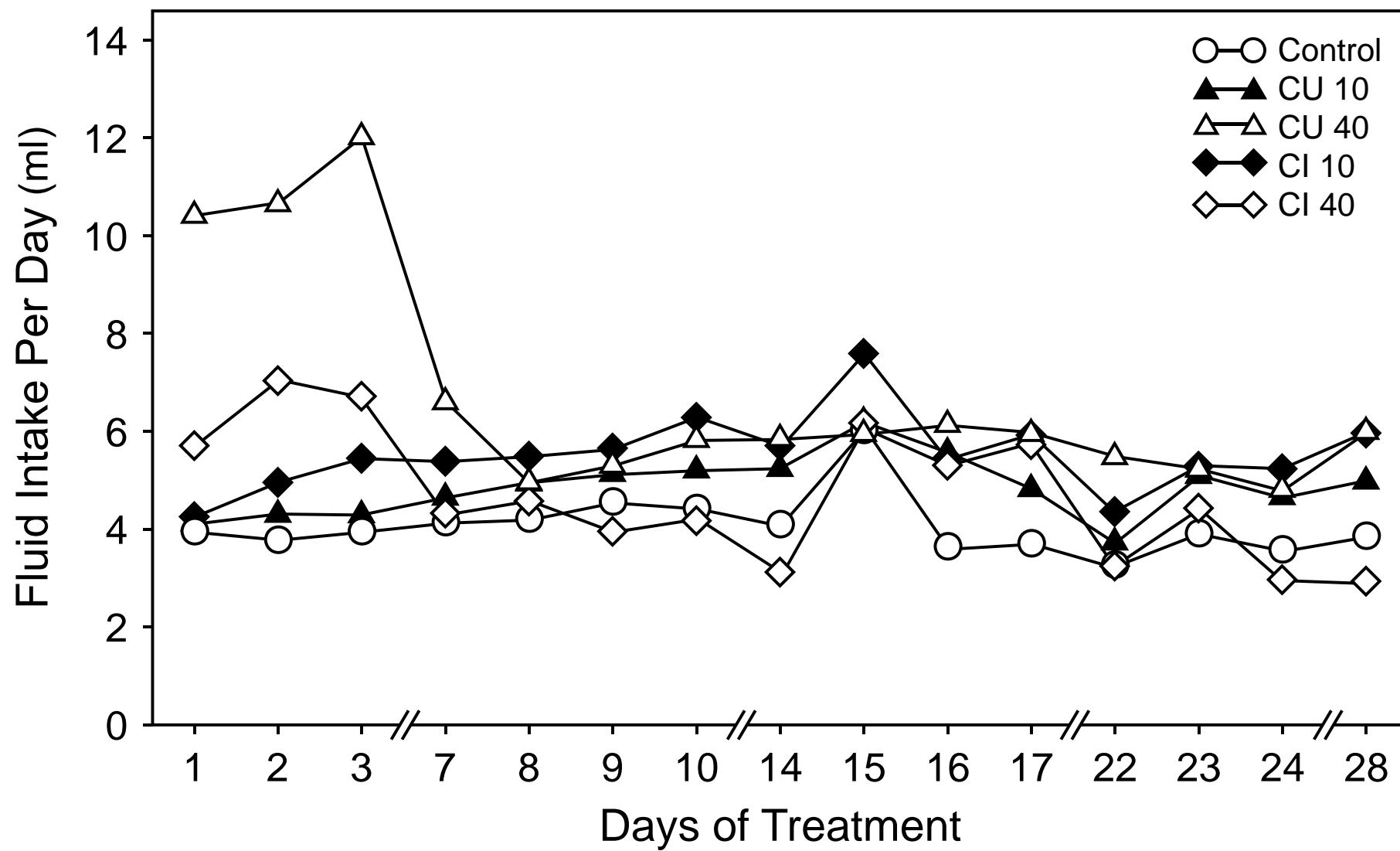

Supplement: S2 Fig — CU; Citrus Unshiu, CI; Citrus Iyo. (PDF) [file pone.0117616.s002.pdf]
